# Ongoing Measles Outbreak in Orthodox Jewish Community, London, UK

**DOI:** 10.3201/eid1910.130258

**Published:** 2013-10

**Authors:** Vanessa Baugh, Jose Figueroa, Joanne Bosanquet, Philippa Kemsley, Sarah Addiman, Deborah Turbitt

**Affiliations:** Public Health England, London, UK (V. Baugh, J. Bosanquet, S. Addiman, D. Turbitt, J. Figueroa);; Homerton University Hospital Foundation Trust, London (P. Kemsley)

**Keywords:** vaccination, measles, infectious disease outbreaks, Judaism, mumps-measles-rubella vaccine, viruses, United Kingdom

**To the Editor:** Measles outbreaks have been reported in Orthodox and ultra-Orthodox Jewish communities across Europe and Israel (*1*–*5*). We describe an ongoing outbreak within the largest European Orthodox Jewish community (including a Charedi population of 17,587), based in London, focused in Hackney (*6*). Vaccination coverage within this community is lower than in the general population of London, causing low herd immunity and outbreaks of vaccine-preventable diseases. Vaccination coverage data within the communities cannot be extrapolated, because membership is not classified as an ethnicity and not collected within health electronic recording systems. However, general practice surgeries in Hackney known to have high proportions of Orthodox Jewish patients have considerably lower vaccination coverage (55%–75% of patients 24 months of age had received measles, mumps, rubella [MMR] vaccine in the 3rd quarter of 2012) compared with the London average (87.3%) (*7*). Health beliefs, family size (the average Charedi household size is 6.3 persons), and underutilization of immunization services contribute to low coverage (*8*,*9*).

The outbreak clinical case definition was taken from Public Health England’s guidance (*10*). It also included membership in the Orthodox Jewish community; residency in the London borough of Barnet, Hackney, or Haringey; and notification during December 20, 2012–March 19, 2013.

After serologic confirmation of measles in the index case-patient, an unvaccinated Orthodox Jewish 4-year-old from Hackney, the case was reported to the Health Protection Team (HPT) on December 20, 2012. The family could not recall having contact with someone with measles. The child attended nursery while infectious; subsequently, cases in 3 secondary patients in the nursery were reported to the HPT. Transmission was observed within households, extended family groups, nurseries, schools, and a camp for Orthodox Jewish teenagers attended by 80 girls (mainly from Hackney) with staff from Italy. Five secondary cases from this camp were reported (in 3 residents of London, 1 resident of Sheffield, and 1 resident of Hertfordshire).

During December 20, 2012–March 19, 2013, a total of 62 notifications of measles cases meeting the case definition were received in residents of Barnet (8 cases), Hackney (47), and Haringey (7). Patients’ ages ranged from 7 months to 27 years (median 7 years). Thirty-four (55%) were female. Fifty-four (87%) had never received an MMR vaccine, and 8 (13%) had received only 1. Three were admitted to the hospital, and 5 were clinically assessed in accident and emergency departments (patients’ ages ranged from 7 months to 4 years).

All case-patients were assessed for risk by the local HPT for vulnerable contacts and source of infection. The HPT provided infection control guidance and an oral fluid testing kit. Sixteen (26%) case-patients could not recall any contact with a person with measles; the remainder stated various epidemiologic links to a case-patient (Figure).

**Figure:**
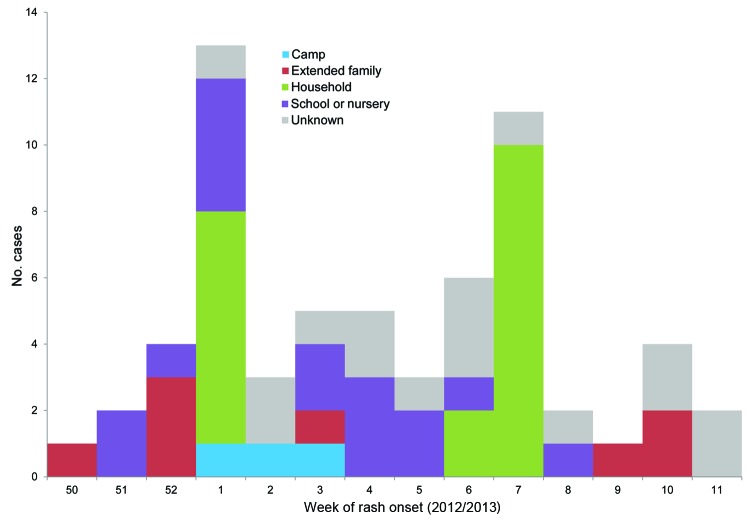
Reported measles cases by week of rash onset and likely source of infection, United Kingdom, 2012–2013.

Forty-two cases have been confirmed (measles IgM detected) by serologic testing (4 cases) or oral fluid (38). One notified case-patient did not have measles IgM on oral fluid testing but had an epidemiologic link to a case-patient and clinical symptoms. They are included in this analysis. Seventeen IgM-positive oral fluid samples were genotyped, and all were D8, currently the most common genotype in the United Kingdom.

One confirmed case was detected in an unvaccinated child from Haringey who was not Orthodox Jewish but was known to have had contact with a case-patient from the community. The child’s illness did not meet the case definition and is not included in this analysis.

In response to the outbreak, active case finding and awareness-raising have been undertaken by the HPT, National Health Service (NHS) public health departments, and community NHS services focused on health and education services and Orthodox Jewish communities. Information letters were sent to the 38 Orthodox Jewish schools and nurseries in Hackney and to attendees of the youth camp. Community NHS vaccination clinics have been maintained to complement standard immunization services offered in general practice surgeries. This includes a Sunday vaccination clinic. Furthermore, community NHS staff provided a vaccination clinic in a secondary school that had an attack rate of 7% (9 cases) at which 9 pupils received 1 MMR vaccine after parental consent. This was the only on-site school vaccination clinic offered; thus, no comparative uptake data are available to supplement our evaluation of the intervention.

Information relating to the outbreak was placed in 2 Orthodox Jewish newspapers and targeted information for families (in English, Yiddish, and Hebrew) has been disseminated. Finally, all 25 HPTs were alerted to this outbreak and the national Public Health England database (HPZone) has been enhanced to capture notifications from Orthodox Jewish communities.

This ongoing outbreak highlights continued health risks in communities with low vaccination coverage. The outbreak has been largely contained within London’s Orthodox Jewish communities, with limited spread outside of the city and to just 1 local non–Orthodox Jewish child. Given the mobility of members, the risk for transmission outside of London is relatively high. The outbreak underscores the need for ongoing evidence-based and culturally appropriate health interventions that seek to improve vaccination coverage.
